# Transgenic APP expression during postnatal development causes persistent locomotor hyperactivity in the adult

**DOI:** 10.1186/1750-1326-7-28

**Published:** 2012-06-18

**Authors:** Shaefali P Rodgers, Heather A Born, Pritam Das, Joanna L Jankowsky

**Affiliations:** 1 Departments of Neuroscience, BCM295, Baylor College of Medicine, One Baylor Plaza, Houston, TX, 77030, USA; 2 Departments of Neurology, Baylor College of Medicine, Houston, TX, USA; 3 Departments of Neurosurgery, Baylor College of Medicine, Houston, TX, USA; 4 Departments of Huffington Center on Aging, Baylor College of Medicine, Houston, TX, USA; 5 Department of Neuroscience, Mayo Clinic Florida, Jacksonville, FL, USA; 6 Present address: Department of Psychology, University of Houston, Houston, TX, USA

**Keywords:** Alzheimer’s disease, Transgenic mouse, Motor hyperactivity, Amyloid precursor protein, APP, Amyloid-β, neurodevelopment, Tetracycline-controllable, Tet-off

## Abstract

**Background:**

Transgenic mice expressing disease-associated proteins have become standard tools for studying human neurological disorders. Transgenes are often expressed using promoters chosen to drive continuous high-level expression throughout life rather than temporal and spatial fidelity to the endogenous gene. This approach has allowed us to recapitulate diseases of aging within the two-year lifespan of the laboratory mouse, but has the potential for creating aberrant phenotypes by mechanisms unrelated to the human disorder.

**Results:**

We show that overexpression of the Alzheimer’s-related amyloid precursor protein (APP) during early postnatal development leads to severe locomotor hyperactivity that can be significantly attenuated by delaying transgene onset until adulthood. Our data suggest that exposure to transgenic APP during maturation influences the development of neuronal circuits controlling motor activity. Both when matched for total duration of APP overexpression and when matched for cortical amyloid burden, animals exposed to transgenic APP as juveniles are more active in locomotor assays than animals in which APP overexpression was delayed until adulthood. In contrast to motor activity, the age of APP onset had no effect on thigmotaxis in the open field as a rough measure of anxiety, suggesting that the interaction between APP overexpression and brain development is not unilateral.

**Conclusions:**

Our findings indicate that locomotor hyperactivity displayed by the tet-off APP transgenic mice and several other transgenic models of Alzheimer’s disease may result from overexpression of mutant APP during postnatal brain development. Our results serve as a reminder of the potential for unexpected interactions between foreign transgenes and brain development to cause long-lasting effects on neuronal function in the adult. The tet-off APP model provides an easy means of avoiding developmental confounds by allowing transgene expression to be delayed until the mice reach adulthood.

## Background

One of the challenges in modeling age-associated diseases in transgenic mice is the need to compress decades of pathogenesis into the two-year lifespan of the laboratory mouse. This is often accomplished by overexpressing disease-associated proteins at high levels from the earliest age possible using strong promoters that are active before birth. Over the past two decades, this approach has yielded several dozen mouse models of Alzheimer’s disease in which transgenic expression of the amyloid precursor protein (APP) is controlled by promoters such as the prion protein, Thy-1 cell surface antigen, or platelet-derived growth factor B chain that drive high-level transgene expression in the adult [1-5]. The inclusion of one or more familial disease-causing mutations alters the enzymatic processing of APP to increase production of amyloid-β (Aβ) 42 peptide (reviewed in [6,7], resulting in the formation of age-associated amyloid deposits similar to those found in human Alzheimer’s patients.

APP transgenic mice also recapitulate the characteristic cognitive symptoms of Alzheimer’s disease. Many APP transgenic models show cognitive decline reminiscent of the human disorder, and several also display non-cognitive symptoms associated with the disease, including anxiety [8-11], aggression [11-17], locomotor hyperactivity [9,18-25] and circadian disturbances [9,13,18,26-28]. In many cases, these non-cognitive phenotypes begin before the onset of amyloid deposition [10-13,15,24,28], and in several models show little change with disease progression [13,21,28] but see [9,10,22,23]. The early appearance and persistent nature of certain behavioral features are not consistent with the gradual accumulation of Aβ, but rather point to a role for the overexpression of mutant APP itself.

Although most of the behavioral and pathological effects of transgene expression are measured in the adult, APP overexpression in several of these models begins during development. Promoters such as the prion protein promoter, PDGF-B, and Thy-1 used to create many common APP transgenic lines are all expressed in the brain by mid-gestation, and have been used to rescue embryonic lethality caused by gene deletion [29-32]. The contribution of developmental APP expression to later pathogenesis has been generally overshadowed by the absence of obvious phenotypic changes until adulthood. Moreover, recent transgenic models have incorporated promoters such as calcium-calmodulin kinase IIα (CaMKIIα) thought to be inactive before birth, thereby avoiding any prenatal effects of APP overexpression [33-35]. Contrary to these assumptions, we discovered an unexpected interaction between postnatal brain development and transgenic overexpression of mutant APP that had behavioral consequences in the adult. Here we show that the severe locomotor hyperactivity displayed by several of our tetracycline-controllable APP transgenic lines can be substantially attenuated by delaying transgene expression until adulthood. Our findings indicate that even phenotypes with relevance to disease may not arise by the same mechanisms in transgenic models as in the human disorder.

## Results

### Delaying transgenic APP overexpression until adulthood attenuates motor hyperactivity

In our original characterization of the tet-off APP transgenic mice, we described severe hyperactivity due to APP overexpression that prevented us from assessing their performance on standard behavioral tasks [35].

**Figure 1:**
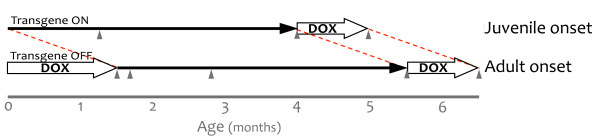
**Using doxycycline to control the onset of transgenic APP expression.** The tet-off APP model uses TTA expressed from the CaMKIIα promoter to control the expression of mutant APP. Under normal conditions, expression from this promoter begins during late embryogenesis and transgenic APP is present at high levels by birth (juvenile onset). Transgene expression can be delayed by rearing the mice on dox, initially transmitted to the pups through their mother’s milk. Dox treatment was used to suppress transgenic APP beginning from shortly after birth (P1-P3) until adulthood (P41-P43). Transgenic APP expression was initiated at 6 wk of age by removing dox from the diet (adult onset). In both sets of mice, bigenic (APP/TTA) and control (TTA) animals were behaviorally evaluated following 7 wk or 4 mo of transgene expression, and again after 1 mo of therapeutic dox treatment to suppress APP expression after the formation of amyloid plaques (grey arrowheads). Adult-onset animals were additionally evaluated immediately after removing dox (0 wk of APP expression), and 1 wk later (1 wk of APP expression) to assess the impact of overproducing APP in the absence of Aβ accumulation. Behavioral testing at equivalent time points (P0 and P7) was not possible in the juvenile-onset animals.

These initial studies monitored animals over several days and revealed abnormally high activity during both phases of the light:dark cycle. Locomotor activity was normalized by lifelong suppression of the transgene, which demonstrated that the effect was not due to insertion site, and was replicated in multiple tet-responsive APP transgenic lines (i.e., line 107, 8–85, and 102, expressing the same transgene and created from the same construct). While seeking a way to examine synaptic plasticity in lieu of cognition with this model, we serendipitously discovered that the hyperactive phenotype could be modified by delaying the onset of transgene expression. This experiment began by rearing APP/TTA mice on doxycycline (dox) until they reached sexual maturity (P1-P3 until P41-P43, Figure 1). This was initially done to generate a series of animals in which the effects of monomeric Aβ, oligomeric aggregates, and deposited amyloid could be studied in acute brain slices from mice that had all reached adulthood. Prior to harvesting the animals for electrophysiological studies, we used the mice to examine whether the aggregation state of Aβ affected motor activity. This simple behavioral evaluation led to the current study in which we describe the interplay between postnatal brain development, transgenic APP overexpression, and motor hyperactivity in the adult.

Three time points were chosen for analysis following onset of transgenic APP: 7 wk after APP induction, just prior to the onset of amyloid formation; 4 mo after APP induction, when a sparse amyloid burden had accumulated; and 1 mo after therapeutic suppression of APP with dox to reduce Aβ production, without affecting pre-existing deposits. We also tested adult-onset mice at two additional time points that could not be examined in juvenile-onset animals because of their age: 0 wk, while animals were still on dox and 1 wk after APP induction, when transgenic APP is fully expressed and Aβ peptide is expected to have reached steady-state levels.

Motor activity was assessed by measuring ambulation – defined as two consecutive photobeam breaks through a frame surrounding the cage - during the first 30 min after being placed into a clean cage. The manipulation was similar to weekly cage changes, using the same shoebox cage bottoms, isolator lids, and wood-chip bedding. Mice were assessed individually during activity monitoring but reunited with their siblings immediately afterwards.

**Figure 2:**
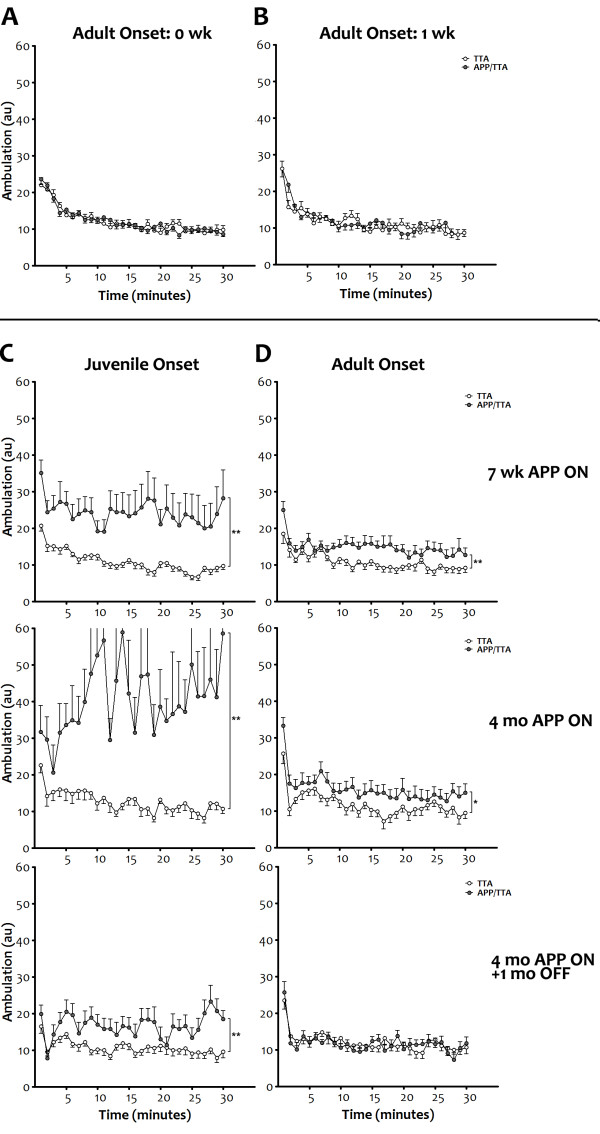
**Juvenile onset of transgenic APP leads to significant motor hyperactivity that can be attenuated by delaying transgene expression. *A, B*** Locomotion was measured using an infrared photobeam system to track activity over time. Activity was identical in control (TTA single-transgenic) and APP transgenic mice (APP/TTA double-transgenic) immediately prior to (0 wk, A; n = 21 TTA, n = 33 APP/TTA) and one wk after the induction of transgenic APP expression in adult-onset mice (1 wk, B; n = 18 TTA, n = 19 APP/TTA). ***C, D*** Hyperactivity was apparent after 7 wk of transgenic APP expression (top row) in both juvenile- (**p < 0.01; n = 23 TTA, n = 20 APP/TTA) and adult-onset mice (**p < 0.01, n = 19 TTA, n = 20 APP/TTA). Both juvenile- (**p < 0.01, n = 12 TTA, n = 8 APP/TTA) and adult-onset mice (*p < 0.05, n = 11 TTA, n = 15 APP/TTA) remain hyperactive with continued transgene expression, although the degree of variability and the magnitude of difference between control and APP transgenic animals is greater with earlier onset (middle row). Suppression of transgene expression for 1 mo after 4 mo of overexpression normalized ambulation levels in APP transgenic mice to that of controls following adult (p = 0.85; n = 15 TTA, n = 14 APP/TTA) but not juvenile-onset (**p < 0.01; n = 15 TTA, n = 13 APP/TTA; bottom row). au: arbitrary units.

**Figure 3:**
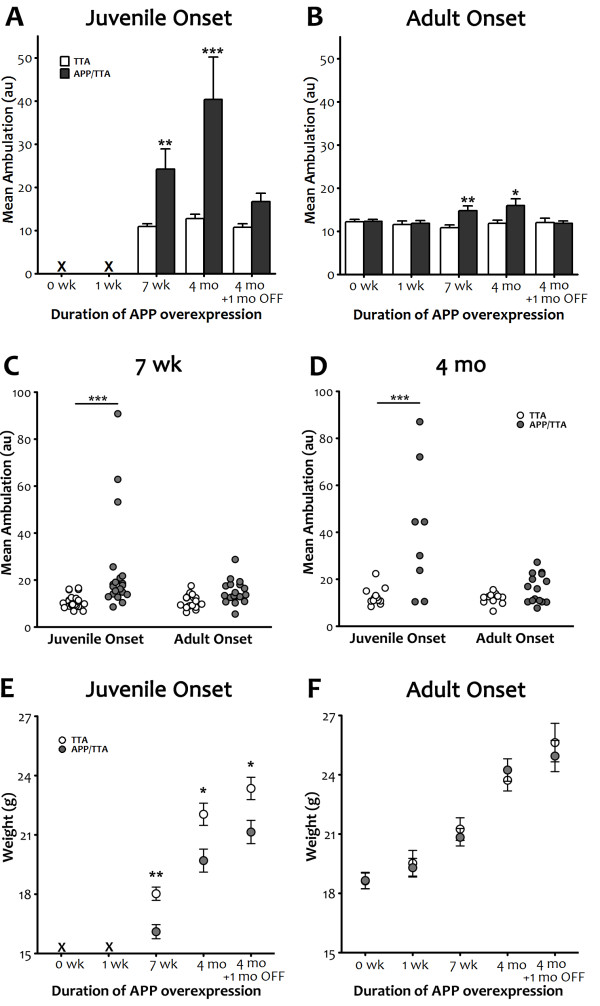
**Delayed expression of transgenic APP reduces motor hyperactivity and normalizes body weight. *A, B*** Comparison of mean ambulation recorded by infrared photobeam monitoring highlights the severity of hyperactivity observed in juvenile-onset APP transgenic animals (a) and the substantial reduction in this phenotype achieved by delaying onset until adulthood (b). Although reduced in magnitude with later onset, post-test analyses identified significant differences between genotypes for both adult- and juvenile-onset at 7 wk and 4 mo (**p < 0.01 for both at 7 wk; ***p < 0.001, for juvenile onset and *p < 0.05 for adult onset at 4 mo). ***C, D*** Replotting the data as individual values illustrates the wide range of locomotor activity seen in mice overexpressing APP from birth and the substantial decrease in both variance and average attained by delaying transgene onset. Individual mean ambulation is shown for juvenile vs. adult onset after 7 wk (C) and 4 mo (D) of transgenic APP expression. Post-test comparisons of groups matched for duration of expression magnifies the difference between genotypes for juvenile onset (***p < 0.001) but diminishes the difference for adult onset (p > 0.05 at both ages). ***E, F*** Mean body weight of APP-overexpressing mice is lower than controls at 7 wk (**p < 0.01) and 4 mo (*p < 0.05) in juvenile onset mice, and this difference persisted even after 1 mo of transgene suppression (*p < 0.05). Delaying transgene onset until adulthood normalized body weight of APP-overexpressing mice at all ages tested.

As expected based on our past work with this line, we found that overexpression of APP from birth resulted in significant hyperactivity in the adult [35]. By 7 wk of age, juvenile-onset APP/TTA mice were substantially more active than their TTA single-transgenic siblings (2-way RM-ANOVA, p < 0.01, Figure 2) and remained hyperactive with continued transgene expression (4 mo, 2-way RM-ANOVA, p < 0.01). On average, juvenile-onset APP/TTA mice were more than twice as active as TTA controls at 7 wk of age (2.2-fold increase, 2-way ANOVA, post hoc p < 0.01, Figure 3) and more than 3 times as active at 4 mo (3.2-fold increase, p < 0.001). While therapeutic suppression of transgenic APP expression between 4 and 5 mo of age (4 mo + 1 mo OFF) substantially reduced the severity of hyperactivity in the APP/TTA mice, it did not eliminate it (2-way RM-ANOVA, p < 0.05), despite past work demonstrating >90% reduction in transgene expression within 2–4 d of dox administration (JLJ, unpublished data). Ambulation remained 1.6-fold higher in APP/TTA mice than in TTA controls after dox treatment, although the difference did not reach statistical significance when averaged over time (2-way ANOVA, post hoc p > 0.05).

Delaying the onset of APP overexpression until adulthood significantly reduced the degree of hyperactivity we observed once transgene expression was initiated. Immediately before (0 wk) and 1 wk after transgenic APP expression began in the adult mice, motor activity in adult-onset APP/TTA mice was identical to that in TTA controls. Behavior did not diverge until 7 wk after adult-onset APP overexpression, when average ambulation was 1.4-fold higher in APP/TTA mice than in TTA controls (2-way ANOVA post hoc p < 0.01). Average ambulation remained constant at 1.4-fold control levels at 4 mo (p < 0.05), but was then completely normalized by 1 mo of therapeutic APP suppression (p > 0.05).

Perhaps as a result of increased motor activity, juvenile-onset APP/TTA mice were noticeably smaller and could be easily distinguished from their littermates. Mean body weights were significantly lower in juvenile-onset APP/TTA mice than in TTA controls at 7 wk (2-way ANOVA, post hoc p < 0.01) and 4 mo (p < 0.05) and this difference persisted after 1 mo of transgene suppression (p < 0.05). In contrast, delaying APP overexpression until adulthood normalized body weight of APP-overexpressing mice at all ages tested (2-way ANOVA, p > 0.05).

### Age of onset does not influence the long-term level of transgene expression

**Figure 4:**
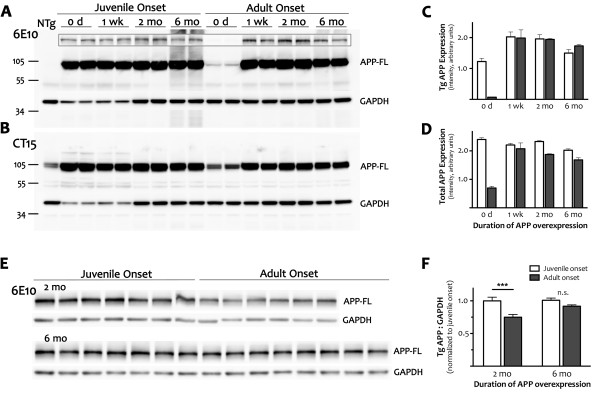
**Adult expression of transgenic APP is independent of age at onset. *A*** Cortical homogenates from juvenile- and adult-onset animals with 0 wk, 1wk, 2 mo or 6 mo of transgenic APP overexpression were immunoblotted with human-specific antibody 6E10 to determine whether timing of onset of APP induction differentially alters forebrain APP expression. The blot was overexposed to visualize the small amount of transgenic leak present in dox-treated mice (0 d, adult-onset). The outlined inset panel shows the same samples loaded with less protein per lane and a shorter exposure time. Glyceraldehyde 3-phosphate dehydrogenase (GAPDH) was co-immunostained as a loading control. ***B*** A separate blot was immunostained with CT15; GAPDH was co-stained as a loading control. As in panel A, the intensity of GAPDH staining was lower in perinatal samples (0 d harvested at P0 and 1 wk harvested at P7) than in adult tissue although similar amounts of protein were loaded in each lane. The same GAPDH expression pattern was replicated with independent samples, and was observed with an unrelated control protein, SOD1 (not shown). ***C***, ***D*** Quantitation of signal intensity for full-length APP from the 6E10 Western blot shown in (A) and the CT15 blot shown in (B). Because GADPH levels change over postnatal development, APP levels are plotted as absolute values rather than relative ratios. Graphs show mean ± SEM, *n = 2* per time point for each age of onset. With just 2 samples per group, these values are intended only to highlight the difference in APP levels at the outset of expression (0 d). ***E*** Immunoblotting with 6E10 in a larger cohort of animals was used to obtain a more accurate measure of full-length APP expression after 2 mo and 6 mo of transgene expression. ***F*** Quantitation of APP intensity at 2 and 6 mo reveals that transgenic protein was lower in adult- than in juvenile-onset mice after 2 mo of expression (***p < 0.001), but reached similar levels at 6 mo (n.s.). Values are calculated relative to GAPDH, which expressed at stable levels in adult mice. Because separate blots were used to quantify APP levels at 2 mo and 6 mo, values for adult-onset were normalized to juvenile-onset at each age. Graph shows mean ± SEM, *n = 6-9* per time point for each age of onset. White bars, juvenile onset; black bars, adult onset.

One possible explanation for diminished hyperactivity in adult-onset mice is that the transgene may have been expressed at lower levels following dox suppression compared to expression levels in juvenile-onset mice. To rule out this possibility, we examined full-length APP levels from juvenile- and adult-onset APP/TTA mice using the transgene-specific antibody 6E10 and the pan-APP antibody CT15 (Figure 4). Expression was tested in forebrain homogenates from P0 and P7 pups, 2 and 6 mo after juvenile onset, immediately prior to adult onset (D0), and 7 d, 2 mo and 6 mo after adult onset.

Transgenic APP was present at birth in juvenile-onset mice. Expression levels at P0 approached (6E10) or were similar to (CT15) those found at later ages. Transgenic APP thus appears earlier than has been reported for endogenous CaMKIIα mRNA [36], but is consistent with the late embryonic onset observed with tet-responsive reporters for TTA activity in the CaMKIIα-TTA transgenic mice (M. Mayford, personal communication). Transgenic APP expression remained high at P7 and throughout adulthood.

In contrast to the immediate presence of transgenic protein in juvenile-onset mice, transgenic APP in adult-onset animals required several days to reach maximal levels following withdrawal of dox. Once induced, expression of transgenic APP in adult-onset mice was similar, although not identical, to the levels observed in juvenile-onset animals. We directly compared the levels of full-length transgenic APP in juvenile- and adult-onset mice following 2 mo and 6 mo of transgene expression by immunoblotting with 6E10. The level of transgenic APP in adult-onset mice was 75.4 ± 4.1% of juvenile-onset after 2 mo of expression (2-way ANOVA, post hoc p < 0.001), and 91.4 ± 2.1% at 6 mo (p > 0.05). Thus, the expression of transgenic APP in adult-onset mice was delayed by several days following dox withdrawal and was approximately 25% lower for several weeks afterwards, but ultimately reached levels similar to those in juvenile-onset animals.

### Delaying expression of transgenic APP slows the onset of amyloid deposition but does not affect subsequent accumulation

**Figure 5:**
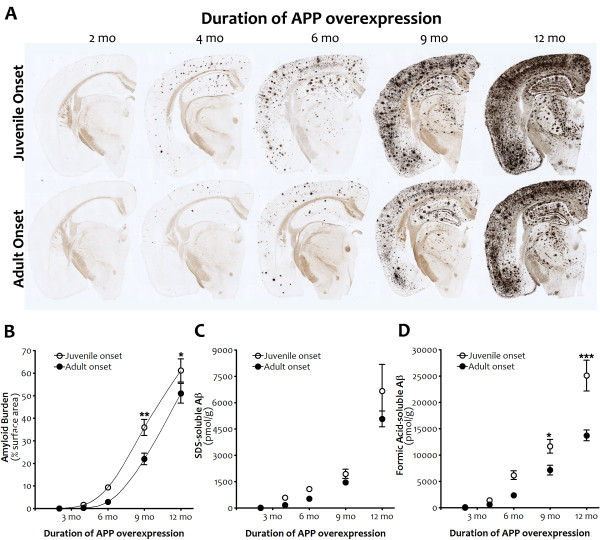
**Amyloid burden and Aβ levels are slightly delayed by adult onset APP overexpression. *A,*** Silver staining shows forebrain amyloid burden after 2, 4, 6, 9 and 12 mo of transgenic APP overexpression following juvenile (top row) or adult onset (bottom row). ***B,*** Quantitation of cortical amyloid burden from silver-stained sections demonstrates that delaying APP overexpression slows initiation of the exponential phase of plaque deposition (p < .001), although once underway the rate of amyloid accumulation is similar in both groups. Post hoc comparisons indicate significant differences in amyloid burden at 9 mo (**p < 0.01) and 12 mo (*p < 0.05) of APP overexpression. n = 3-4 per genotype at each time point. ***C, D*** Biochemical measures of Aβ concentration parallel histological measures of amyloid load. After equivalent durations of APP overexpression, adult onset is associated with lower levels of both SDS- and FA-soluble Aβ (Aβ 40 plus 42; p < 0.05 (SDS), p < 0.0001 (FA)). Additionally, the rate of change in FA-soluble Aβ is slower following adult onset (p < 0.001). Post hoc comparisons indicate significant differences in FA-soluble Aβ at 9 mo (*p < 0.05) and 12 mo (***p < 0.001) of APP overexpression. Graphs show mean ± SEM. n = 6-8 per genotype at each time point. Open symbols, juvenile onset; closed symbols, adult onset.

Another potential explanation for reduced hyperactivity in the adult-onset mice was that the diminished transgene expression following dox withdrawal did not yield enough Aβ to form either soluble aggregates or amyloid deposits within the 4 mo window of our experiments. To rule out this possibility, we measured the percent of cortical surface area covered by amyloid deposits in silver-stained sections from juvenile- and adult-onset APP/TTA mice at 2, 4, 6, 9, and 12 mo after APP induction. We found that the exponential phase of plaque deposition was delayed following adult onset of APP expression (2-way ANOVA, p < 0.001, Figure 5), but once started, the rate of amyloid accumulation was similar in both groups of mice (p > 0.05). Consistent with this overall shift in the curve, post hoc comparisons reflected higher amyloid levels at 9 and 12 mo of APP overexpression (p < 0.01 and p < 0.05, respectively) in juvenile- compared to adult-onset mice.

Biochemical measures of Aβ concentration paralleled histological measures of plaque load. The accumulation of Aβ in adult-onset mice eventually reached the same milestones as juvenile-onset animals, but did so at a slightly later age. Two-way ANOVA revealed that overall, adult-onset mice harbored lower levels of SDS-soluble (p < 0.05) and formic acid-soluble (p < 0.0001) Aβ (40 + 42) than juvenile-onset animals. Post hoc comparisons indicated higher levels of formic acid-soluble Aβ in juvenile-onset mice at 9 and 12 mo of APP overexpression (p < 0.05 and p < 0.001, respectively).

To obtain an estimate of the temporal shift in amyloid formation caused by delaying APP overexpression, we fit the curves to polynomial equations and solved for age at varying levels of amyloid burden. The age at which adult-onset mice reached 20%, 30%, and 40% surface area was on average 6 wk later than juvenile-onset (range: 1.41-1.45 mo). This time is coincidentally the same as the delay in APP overexpression until adulthood, but because the data are already corrected to depict amyloid load at equivalent durations of transgene expression, this reflects an additional delay that was likely caused by the initial lag in transgene onset and slight reduction in APP expression levels during the first few months afterwards.

### Locomotor hyperactivity is independent of amyloid burden

Our discovery that amyloid burden was consistently lower in adult-onset mice than in juvenile-onset mice raised the possibility that ambulation differences we observed in mice matched for duration of APP overexpression were due simply to a difference in plaque load and not to a sustained change in motor behavior. To rule out this possibility, we generated 3 new cohorts of animals. One set of animals was allowed to overexpress APP from birth and was tested at 6 mo of age. The other two groups of mice were reared on dox until 6 wk of age; one group was tested 6 mo later and the other 8 mo later. These three groups allowed us to test the role of APP onset and amyloid burden in motor hyperactivity by comparing behavior in mice with 1) equal duration of APP overexpression but different amyloid burden (6 mo juvenile onset vs. 6 mo adult onset), 2) equal amyloid burden but different age of onset (6 mo juvenile onset vs. 8 mo adult onset), and 3) equal age of onset but different amyloid burden (6 mo adult onset vs 8 mo adult onset). These ages were chosen to represent a stage of disease more likely to be used for cognitive testing than the beginning phase of pathology we studied in our first experiments. Because our laboratory moved from Caltech to Baylor between completing the original ambulation studies and beginning these, we were restricted to measuring locomotor activity by the less expensive and more common open field assay. However, this offered the opportunity to confirm our phenotype with an independent behavioral test, and to assess locomotor activity in a format more likely to be used for screening prior to cognitive testing.

**Figure 6:**
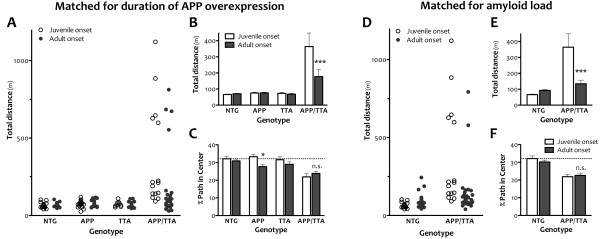
**Delaying the onset of APP overexpression attenuates hyperactivity but not anxiety in the open field assay.** An independent cohort of animals was tested by open field assay at a single time point following 6 mo of APP overexpression. ***A*** A fraction of APP/TTA mice in both juvenile and adult onset groups show extreme hyperactivity, traveling >500 m in 30 min (5/15 juvenile onset, 4/26 adult onset), but most cover distances closer to the average of the other genotypes. ***B*** While both adult- and juvenile-onset APP/TTA mice travel greater average distances than other genotypes (p < 0.0001), delaying the onset of transgene expression significantly diminished hyperactivity compared to juvenile onset (***p < 0.001). ***C*** Juvenile-onset APP/TTA mice travel a smaller fraction of their total path within the center of the open field arena than control mice (p < 0.001 vs. NTG and APP, p < 0.01 vs. TTA). This difference is not normalized by delaying the onset of transgene expression. The only genotype for which dox rearing significantly altered path in center was APP (*p < 0.05). ***D*** A separate cohort of NTG and APP/TTA mice was reared on dox until 6 wk of age and then tested in the open field 8 mo later. Data from the 8 mo adult-onset mice are plotted alongside 6 mo juvenile-onset mice taken from panel A to provide a comparison of open field activity in mice matched for amyloid load rather than duration of APP overexpression. Again, delaying transgene onset reduces the fraction of mice displaying extreme hyperactivity (2/37 adult onset, 5/15 juvenile onset). ***E*** Average distance traveled is significantly reduced by delaying transgene onset (***p < 0.001). While open field activity of juvenile-onset APP/TTA mice is significantly higher than their NTG siblings (p < 0.05), adult-onset APP/TTA mice are no different than age-matched NTG controls (p > 0.05). ***F*** Despite significant attenuation of locomotor hyperactivity, delaying APP overexpression does not alter anxiety measured as % path in the center of the open field arena.

Consistent with our earlier results, overexpression of transgenic APP from birth leads to significant hyperactivity in the open field at 6 mo of age (ANOVA, p < 0.0001, Figure 6). Juvenile-onset APP/TTA mice traveled a greater average distance during the 30 min test than any other genotype (post hoc p < 0.001 vs. NTG, APP, and TTA). Also consistent with our earlier findings, delaying the onset of APP overexpression until adulthood significantly attenuated, but did not completely extinguish, this hyperactivity. Adult-onset APP/TTA mice with 6 mo of APP overexpression traveled, on average, a significantly greater distance than other genotypes (ANOVA, post hoc p < 0.05 vs. NTG, APP, and TTA), but significantly less than juvenile-onset APP/TTA mice with the same duration of APP overexpression (2-way ANOVA, post hoc p < 0.001). This comparison demonstrates that our findings from open field testing were consistent with those obtained by infrared activity frames, and that differences in motor activity present earlier in the progression of pathology persisted to later stages.

Interestingly, data from both juvenile- and adult-onset APP/TTA mice were bimodally distributed. A minor fraction of the animals ran distances > 500 m, while the remainder ran < 250 m. Delaying transgene onset reduced the fraction of mice exhibiting this extreme behavior (5/15 or 33% following juvenile onset, 4/26 or 15% following adult onset). In some experimental settings, mice with such high activity levels might be considered outliers. When these animals are removed from analysis, the difference between juvenile-onset APP/TTA mice and their siblings remained (ANOVA, post hoc p < 0.001 vs NTG, APP, and TTA), while the difference between adult-onset APP/TTA mice and their siblings is lost (p > 0.05). This indicates that a small number of highly active animals drove the difference between genotypes in adult-onset mice, and that when these animals are identified and removed, the remaining cohort is similar in motor activity to single- and NTG controls. This was not true in juvenile-onset mice where the majority of APP/TTA mice are behaviorally distinct from their siblings.

We next compared open field activity between juvenile- and adult-onset mice that were matched for amyloid load rather than duration of APP overexpression. For this comparison, we tested APP/TTA mice that were reared on dox until 6 wk of age and assayed in open field 8 mo later against APP/TTA mice that were never on dox and tested at 6 mo of age. Based on the measurements shown in Figure 5, cortical amyloid burden in adult-onset mice at this age is approximately 14.3%, up from 2.9% two months earlier, and a better match for the 9.3% burden found in juvenile-onset mice at 6 mo of age. Even with slightly more amyloid and 2 mo longer exposure to transgenic APP, adult-onset APP/TTA mice following 8 mo of APP over-expression were significantly less active in the open field than juvenile-onset mice at 6 mo (2-way ANOVA, post hoc p < 0.001). Fewer APP/TTA mice displayed extreme hyperactivity in the adult-onset group (2/37 or 5% vs. 5/15 or 33%), and as at 6 mo, when these highly active animals were removed from analysis, the remaining adult-onset APP/TTA mice were no different than NTG (ANOVA, p > 0.05). Further, there was no difference in open field activity between the two groups of adult-onset APP/TTA mice tested after 6 and 8 mo of APP overexpression (2-way ANOVA, post hoc p > 0.05), suggesting that although amyloid burden increased almost 5 fold during that time, locomotor activity did not. Moreover, there was no difference in total distance traveled by NTG mice under the two conditions, indicating that the 3.5 mo age difference likely did not play a significant role normalizing activity in the adult-onset APP/TTA mice.

### Delaying the onset of transgenic APP expression does not normalize all behavioral phenotypes

Open field testing also provided a coarse measure of anxiety, another common phenotype in APP transgenic mice. Highly anxious or neophobic animals tend to remain close to the walls of the chamber when examined in the open field test, and the severity of this phenotype can be roughly assessed by calculating how much time the animal spends, or how far it travels, in the center of the arena as a fraction of the total exploration time or distance [37,38]. Compared to single transgenic and NTG controls, 6 mo old juvenile-onset APP/TTA mice traveled a significantly smaller fraction of their total path length in the center of the arena (ANOVA, p < 0.001). While 32.1 ± 1.5% of the path traveled by NTG mice was spent in the center area, only 21.9 ± 1.8% was spent in the center by APP/TTA mice (post hoc p < 0.001 vs. NTG and APP, p < 0.01 vs. TTA). Delaying transgene onset until adulthood did not abate thigmotaxis in the open field. Six months after adult onset, APP/TTA mice traveled significantly less of their total path in the center of the arena than single transgenic and NTG controls (ANOVA, p < 0.001). The percentages were similar to juvenile-onset values: 30.9 ± 1.0% of the path traveled by dox-reared NTG was spent in the center area, while only 23.8 ± 1.2% was spent in the center by APP/TTA mice (post hoc p < 0.001 vs. NTG, p < 0.05 vs. TTA). Open-field thigmotaxis persisted at 8 mo after adult onset, when APP/TTA mice traveled 23.3 ± 1.4% of their path in the center vs. 30.4 ± 1.1% in age-matched dox-reared NTG animals (2-way ANOVA, p < 0.001). Like total distance traveled, the percent path in center did not differ between 6 and 8 mo after transgene onset, indicating that although amyloid burden increased, anxiety did not. Although for now limited to this single measure of emotionality, our findings suggest that delaying the onset of APP overexpression does not unilaterally affect all behavioral phenotypes.

## Discussion

During our initial characterization of the tet-off APP transgenic lines in 2005 we noted motor hyperactivity during both light and dark phases of the light cycle that was severe enough to preclude cognitive analysis [35]. Here we show that this motor hyperactivity can be substantially attenuated by delaying overexpression of the mutant protein until adulthood. This finding suggests that the postnatal brain is susceptible to APP overexpression in a way that the mature brain is not. Exposure to transgenic APP during postnatal development had long-lasting effects on adult brain function that were exacerbated by continued expression of mutant APP. However, motor hyperactivity in juvenile-onset mice persisted at a modest level even after therapeutic suppression of transgenic APP, indicating that once established, the behavior is no longer dependent on continued APP overexpression to be maintained.

Our findings are consistent with past reports describing motor hyperactivity in several other lines of APP overexpressing mice. Significant elevations in open field activity and home cage ambulation have been described in both Tg2576 and CRND8 models [9,13,19-22]. Both of these transgenic lines are controlled by the prion protein promoter [2,5], which in other transgenic models is active in the brain before birth [30]. As with the CaMKIIα promoter used here, overexpression of transgenic APP from the prion protein promoter would be expected by the second postnatal week when the nigrostriatal pathway undergoes considerable structural maturation [39]. Endogenous APP plays a role in shaping neuronal morphology and establishing synaptic connections during development, and loss of APP through targeted deletion in vivo or by shRNA knockdown in vitro results in neurite overgrowth and excessive, but poorly aligned, pre- and post-synaptic structures [40-43], reviewed in [44]. Paradoxically, overexpression of APP can cause similar changes in immature neurons, increasing neurite outgrowth and the density of dendritic spines [45,46]. In vivo, the effect of APP overproduction on neuron morphology is often confounded by the concurrent accumulation of Aβ in models expressing FAD mutations. However, overexpression of wild-type APP – absent the Aβ overproduction of FAD variants – leads to increased synapse density both in the brain and in the periphery [47-49]. Taken together, changes in APP expression (both up and down) can affect neuronal outgrowth and synapse formation in the brain and would be present at the right time and place to modify neural circuitry in the maturing nigrostriatal pathway.

While delaying the onset of transgenic APP until adulthood reduces the severity of motor hyperactivity, it does not completely eliminate the behavior. Slight but significant increases in ambulation developed by 7 wk of APP overexpression and persisted at the same level when tested at 4 mo. However, unlike the residual hyperactivity that remained following therapeutic APP suppression in mice with juvenile transgene expression, 1 mo of dox treatment completely normalized motor activity in adult-onset mice. This indicates that in adult-onset mice, motor hyperactivity was dependent on the presence of transgenic APP. Consistent with this, past work has shown that infusion of Aβ into the brains of wild-type mice can acutely modulate dopamine release [50,51]. However, the initial lag between induction of transgenic APP by 1 wk after dox withdrawal (Figure 5) and the appearance of hyperactivity at 7 wk (Figure 3) suggests that the behavioral phenotype was not a direct effect of APP overproduction. Instead, the delay between transgene induction and the appearance of hyperactivity suggests that the effect may be indirect, perhaps through functional changes in the nigrostriatal pathway, such as modulation of dopamine receptor sensitivity, alterations in dopamine production, or transmitter uptake. Consistent with this possibility, APP/PS1 transgenic mice show altered tyrosine hydroxylase immunoreactivity and diminished size of dopaminergic neurons within the susbstantia nigra long before the appearance of amyloid [52]. Alternatively, the delay between transgene induction and behavioral changes in the adult-onset mice (i.e., activity after 1 wk of APP over-expression was identical to NTG but by 7 wk became significantly elevated) may suggest dependence on the accumulation of an aggregated, but reversible, form of Aβ. Further experiments, such as in vivo microdialysis to measure striatal dopamine release, or secretase inhibition to prevent Aβ production during continued APP expression, will be needed to distinguish these possibilities.

Our study further demonstrates that the motor phenotype can be modulated independently from amyloid pathology. Hyperactivity began prior to amyloid formation, in both juvenile- and adult-onset mice. In both groups, cortical plaque burden was < 0.05% after 2 mo of APP overexpression, yet ambulation was already elevated a week earlier at 7 wk. Hyperactivity worsened after amyloid formation in juvenile-onset mice, but remained unchanged from pre-deposit levels in adult-onset animals. The dissociation between plaques and motor activity was most pronounced following therapeutic suppression of transgenic APP. Dox treatment begun after the appearance of amyloid deposits has been shown to stabilize plaque burden, preventing further accumulation but not promoting clearance [35,53]. APP suppression completely normalized motor activity in adult-onset mice and significantly reduced hyperactivity in juvenile-onset animals, despite the continued presence of amyloid in both.

There are three caveats to our interpretation that are worth noting. The first is that as late as 2 mo after onset, the level of transgene expression in dox-reared animals is not identical to dox-naïve. Although the level of APP overexpression following adult-onset ultimately matches that of juvenile-onset mice, it starts off approximately 25% lower. If the absolute level of transgenic APP and not the age at which it is expressed is the critical factor in determining hyperactivity, we may have mistaken a concentration difference for an effect of timing. That other APP transgenic models with lower expression levels also display motor hyperactivity suggests this is not the case. There is likely a minimum level of mutant APP expression needed to evoke this phenotype, as a companion line of tetO-APPswe/ind mice generated at the same time as the line used here, but which produced approximately one-quarter the amount of transgenic APP, did not develop motor hyperactivity (line 70, data not shown). The adult-onset mice studied here expressed well in excess of this minimum, but the fact that they expressed at lower levels for several months and started almost a week later than juvenile-onset animals (once embryonic expression and the delay between dox withdrawal and transgene onset are taken into account) meant that they also developed amyloid at a later age. If the absolute amyloid levels were a critical factor in determining hyperactivity, a better experimental design would have matched adult- and juvenile-onset animals for plaque burden rather than duration of APP expression. In most of our comparisons, amyloid load was lower in adult-onset animals (0.26 ± 0.038% vs. 1.6 ± 0.18% after 4 mo of transgenic APP expression, 2.92 ± 0.49% vs. 9.31 ± 0.67% at 6 mo). However, under conditions that matched amyloid load rather than duration of APP overexpression (14.3% at 8 mo after adult onset (calculated), and 9.3% at 6 mo after juvenile onset (actual)), hyperactivity was still greater in mice that expressed APP from birth. This leads into the final caveat to our interpretation of the data. In several of our experiments, the magnitude of the difference in open field ambulation and ambulation is driven by a small number of outliers. While the size of the effect shrinks when these animals are removed from analysis, the significance remains. Thus, the behavioral impact of transgenic APP during postnatal development is fully penetrant, albeit highly variable in scale.

## Conclusions

Transgenic models based on APP overexpression have been extraordinarily successful in recapitulating the late-onset pathology of Alzheimer’s disease within the two-year lifespan of the laboratory mouse. Their widespread use makes the artificial nature of these models easy to look past, but forcing amyloid formation to occur in 1 year or less generally requires expression of mutant protein at levels several-fold higher than normal using promoters that are active in temporal and spatial patterns distinct from the endogenous gene. Here we show that developmental overexpression of APP can cause lasting changes in the behavior of adult animals that persist even after the transgene is suppressed. While we suspect only a limited number of behavioral phenotypes observed in APP transgenic mice arise from developmental interactions, our findings are a reminder of the inherent limitations of our models. Given that many behavioral tests used to examine learning and memory in rodents rely on controlled locomotor activity (i.e., fear conditioning, Y-maze, object recognition, etc.), hyperactive animals may be mistakenly considered cognitively impaired, and successful therapies may simply normalize motor function. The tet-controllable nature of our model offers an easy means of limiting this issue and opens the possibility of disentangling the developmental consequences of APP overexpression from adult-onset changes. The presence of excess APP in the adult may yet have additional consequences for neural function unrelated to disease that remain a constant caveat when working with model systems. Provided we acknowledge their limitations and apply due caution, transgenic mice offer an unparalleled platform for testing hypotheses and refining how we think about disease.

## Methods

### Mice

Male and female tetracycline-responsive APP transgenic mice were derived from the intercross of line 102 tetO-APPswe/ind expressing a chimeric mouse APP with a humanized Aβ domain and the Swedish and Indiana mutations (MMRRC #34845, distributed via Jackson Laboratories [35] with CaMKIIα-tTA line B expressing the tetracycline transactivator under control of the CaMKIIα promoter (Jackson Laboratories #3010 [54]) to generate APP/TTA double transgenic males for breeding. Both lines had been backcrossed to C57BL/6 J for >20 generations prior to the intercrossing. The APP/TTA males were then mated with wild-type C57BL/6 J females to produce offspring for analysis. All pups were fostered to outbred ICR females for rearing to improve viability and avoid cannibalism of pups common in inexperienced C57BL/6 J breeders [55]. Equal numbers of male and female offspring were used for study. Animal experiments were performed under protocols that were reviewed and approved by the Institutional Care and Use Committee at either California Institute of Technology or Baylor College of Medicine.

### Doxycyclince treatment

One half of the animals used for study were reared on doxycycline to suppress APP overexpression for the first 6 weeks of life. This was initially done by feeding dox chow to nursing females who transmitted the drug through their milk to the pups, and then continued by maintaining the pups on dox chow after weaning. At 6 weeks of age, the mice were returned to regular (unmedicated) chow to initiate transgenic APP expression. Dox was not initiated until between P1 and P3 to avoid problems associated with continuous suppression. Past work has shown mice receiving dox from conception to adulthood express the transgene at lower levels than expected when the drug is removed [56]. This is consistent with DNA modification such as methylation of silent regions, and has been exploited in past studies as a way to generate transgenic mice from the same line with 30-60% of the expected level of expression.

During the course of this study, and coinciding with our move from California Institute of Technology to Baylor College of Medicine, we discovered that transgenic APP expression in line 102 could be suppressed equally well with 50 mg/kg doxycycline as with the original concentration of 200 mg/kg (JLJ, HAB, unpublished data). Animals reared at Caltech and tested by infrared beam activity frames described below were therefore treated with the original chow formulated at 200 mg/kg doxycycline (F4845; BioServe, Frenchtown, NJ) while animals reared at Baylor and used for open field assay, biochemistry, and histology were treated with chow containing 50 mg/kg doxycycline (F5903), both formulated into a base diet of Purina 5001 rodent chow.

### Activity monitoring

Animals were separated into clean cages immediately before the start of each experiment. The cages were placed inside photobeam frames to monitor the animals’ movement over time (San Diego Instruments, San Diego, California, United States). Experiments were started midway through the light phase of the day, and data was collected in 1 min bins for 30 min. Testing rooms were maintained on the same 13:11 h day:night cycle as the main animal housing areas and were closed to entry during the experiment.

### Open field assessment

Each mouse was placed in the center of an open-top white acrylic box (20 in. x 20 in. x 9 in.) and allowed to explore freely for 30 minutes. Movement was recorded and analyzed using the ANY-maze Video Tracking System (Stoelting Co., Wood Dale, IL). The center of the arena was defined as a square occupying 1/3 of the total area, and the distance traveled in this field was calculated as a percentage of total distance traveled in the arena.

### Tissue harvest

Animals were perfused with PBS and the isolated brains hemisected along the midline. One hemisphere was frozen for biochemical analyses; the other hemisphere was immersion-fixed for 48–96 hr at 4°C in 4% paraformaldehyde.

### ELISA

Hemi-forebrain samples were prepared for analysis of Aβ levels by two-step sequential extraction using 2% SDS followed by 70% formic acid (FA) as previously described [57]. Aβ levels were determined by end-specific sandwich ELISAs using mAb 2.1.3 for capture (human Aβx-42 specific) and HRP-conjugated mAb Ab9 (human Aβ1–16 specific) for detection, or mAb Ab9 for capture and HRP-conjugated mAb 13.1.1 (human Aβx-40 specific) for detection [58,59]. All values were calculated as pmol per g based on the initial weight of brain tissue.

### Immunoblotting

The 2% SDS homogenates prepared for ELISA were diluted 1:1 with 2x-concentrated high-detergent RIPA buffer minus SDS (2xPBS, 1% deoxycholate, 1% NP40, 5 mM EDTA, plus protease inhibitors). Protein concentration was measured by BCA assay for each sample, and approximately 50 μg of the resulting homogenate was separated on 10.5-14% Tris-glycine or 10-20% Tris-tricine gels for quantitation of APP levels (BioRad Criterion). After transfer to nitrocellulose (Schleicher and Schuell Optitran), blots were probed with mouse anti-human APP/Aβ antibody 6E10 (1:2000 or 1:5000, Signet #9300-02), or with rabbit anti-APP C-terminal fragment antibody CT15 (1:2000, kind gift of Eddie Koo), either in conjunction with or followed by incubation with chicken anti-GAPDH polyclonal antibody (1:5000, Millipore Ab2302). Binding was detected with HRP-labeled secondary antibodies and developed with ECL reagent (Millipore Immobilon). Chemiluminescence was measured with a Fuji LAS-4000 mini CCD system and quantified using Multi Gauge software.

### Histology

Immersion-fixed hemibrains were cryoprotected and embedded 40 per block in a solid matrix. Coronal sections were cut from the frozen block at 35μm (MultiBrain^TM^ processing by NeuroScience Associates, Knoxville, TN) and stored in cryoprotectant at −20°C until use. Amyloid was detected from a 1 in 12 series of sections using the Campbell-Switzer silver stain. A detailed protocol for this stain can be found online at the NeuroScience Associates website http://www.neuroscienceassociates.com/Documents/Publications/campbell-switzer_protocol.htm.

### Quantitation of histology

Sections were analyzed using a macro written for AxioVision 4.7. Color thresholds were used to identify amyloid plaques in high-resolution digital scans of the stained slides. Background staining and shading artifacts were manually excluded from the analyses. The region of interest was specified by tracing the cortex of the corresponding section and the area of pixels above threshold computed relative to the total area for the region of interest. Four sections spaced at 420 μm intervals spanning approximately −1.3 to −2.7 mm from bregma [60] were analyzed for each animal.

### Statistics

All statistics were done using Prism 5.0. Comparisons of multiple groups were done by one-way ANOVA or two-way ANOVA followed by Tukey or Bonferroni post hoc test, respectively. Comparisons limited to two groups were done by Student’s t-test with Welch’s correction for unequal variances where appropriate. All graphs display group mean ± SEM.

## Competing interests

The authors declare that they have no competing interests.

## Authors’ contributions

SPR carried out open field experiments, harvested animals, quantified amyloid burden from histological samples, and performed statistical analyses; HAB harvested animals and assisted with sample preparation for biochemical analyses; PD performed ELISA measurement of Aβ concentration; JLJ designed the experiment, conducted ambulation tests, performed immunoblot analyses, analyzed data, and wrote the manuscript. All authors read and approved the final manuscript.
